# *Chlamydia* Uses K^+^ Electrical Signalling to Orchestrate Host Sensing, Inter-Bacterial Communication and Differentiation

**DOI:** 10.3390/microorganisms9010173

**Published:** 2021-01-15

**Authors:** Susan C. Andrew, Maud Dumoux, Richard D. Hayward

**Affiliations:** 1 Institute of Structural and Molecular Biology, University College London & Birkbeck, Malet Street, London WC1E 7HX, UK; susan.clements@mintdiagnostics.com; 2 Mint Diagnostics, Unit 1093b Kent Science Park, Sittingbourne ME9 8GA, UK; 3 Rosalind Franklin Institute, Harwell Campus, Didcot OX11 0DE, UK; 4 Department of Pathology, University of Cambridge, Tennis Court Road, Cambridge CB2 1QP, UK

**Keywords:** *Chlamydia*, host-pathogen interactions, cell-to-cell communications and community

## Abstract

Prokaryotic communities coordinate quorum behaviour in response to external stimuli to control fundamental processes including inter-bacterial communication. The obligate intracellular bacterial pathogen *Chlamydia* adopts two developmental forms, invasive elementary bodies (EBs) and replicative reticulate bodies (RBs), which reside within a specialised membrane-bound compartment within the host cell termed an inclusion. The mechanisms by which this bacterial community orchestrates different stages of development from within the inclusion in coordination with the host remain elusive. Both prokaryotic and eukaryotic kingdoms exploit ion-based electrical signalling for fast intercellular communication. Here we demonstrate that RBs specifically accumulate potassium (K^+^) ions, generating a gradient. Disruption of this gradient using ionophores or an ion-channel inhibitor stalls the *Chlamydia* lifecycle, inducing persistence. Using photobleaching approaches, we establish that the RB is the master regulator of this [K^+^] differential and observe a fast K^+^ exchange between RBs revealing a role for this ion in inter-bacterial communication. Finally, we demonstrate spatio-temporal regulation of bacterial membrane potential during RB to EB differentiation within the inclusion. Together, our data reveal that *Chlamydia* harnesses K^+^ to orchestrate host sensing, inter-bacteria communication and pathogen differentiation.

## 1. Introduction

*Chlamydia trachomatis* are obligate intracellular bacteria responsible for the most prevalent bacterial sexually transmitted infection worldwide [1]. *Chlamydiae* undergo a biphasic developmental cycle, during which they are dependent upon interactions with the host cell. Infectious elementary bodies (EBs) invade cells and reside within a modified vesicle. After traffic toward the centrosome, EBs differentiate into reticulate bodies (RBs) that replicate in a membrane-bound compartment named the inclusion. Subsequently, RBs differentiate into EBs, which are released into the extracellular environment. In addition to these two forms, transient persistent bodies (PBs) form in cell culture models, which can revert to RB and complete the infection cycle when favourable conditions are restored. It is suggested that the persistence observed in vitro is the mechanism responsible for the re-emergence of infection in patients after years of apparent remission [2,3], as well as inflammation due to the latent presence of *Chlamydia* antigens and transmission of infection into secondary organs [4,5]. Persistence is triggered by a number of stressors such as antibiotics [6], the absence of amino acids [6,7], the presence of certain cytokines [8,9], endogenous danger signals such as ATP [10] or iron ion restriction [11].

Since organisms like *Chlamydiae* grow as a bacterial population within a cellular compartment, there is a potential need for communication within the bacterial community as well as between the bacteria and the host cytoplasm. It is known that *Chlamydiae* use a variety of effectors to manipulate or hijack host processes [12]. However, this communication is unilateral and does not satisfy the requirement for the bacteria to sense conditions in the host. In parallel, while it is clear that the chlamydial community prepare processes such as egress [13], to date there is no mechanistic explanation for this. Interestingly, bacterial communities forming a biofilm are able to transmit information using waves of potassium ions (K^+^), and therefore synchronise the modulation of their membrane potential, a mode of communication influenced by nutrient depravation [14]. Moreover, it is well known that eukaryotic systems modulate membrane potential through K^+^ flux to transduce information [15,16]. It is therefore tempting to speculate that *Chlamydia* might exploit K^+^ fluxes to sense the host cell and to synchronise the activity of the intracellular community. Indeed, multiple examples demonstrate that variations in [K^+^] in the cytoplasm of infected cells can influence the fate of pathogens. For example, *Salmonella enterica,* uses a K^+^ transport system to regulate host cell invasion [17], the infectivity of *Plasmodium* sporozoites exposed to K^+^ concentrations equivalent to intracellular environments increases by 4 to 10-fold [18] and loss of cytoplasmic K^+^ triggers the egress of intracellular *Toxoplasma gondii* [19].

In *Chlamydia*, although sequence homology suggests the presence of K^+^ channels in the genome, the only role for K^+^ demonstrated experimentally during the bacterial lifecycle is for the maturation of cytokines [20]. In parallel, a decrease in cytoplasmic [K^+^] during infection using high multiplicities of infection (MOI) of *C. psittaci* (MOI = 100) has been shown at a late time point [21], although this was dependent on high bacterial load, since it was undetectable at MOI = 10 [21]. Therefore, the K^+^ distribution and variation during *Chlamydia* infection remain unexplored.

Using a new generation of fluorescent affinity K^+^ probes, we demonstrate that RBs specifically accumulate K^+^, in contrast to the EBs. This accumulation generates a [K^+^] differential between the RB and the cytosol as well as the RB and the inclusion lumen. Using ionophores and ion channel inhibitors, we demonstrate that changes in cytoplasmic [K^+^] in the host induce bacterial persistence. Additionally, we show that persistence induced by a range of stimuli induces an abnormality in the [K^+^] ratio between the bacteria and the cytosol or the inclusion lumen demonstrating the importance of the [K^+^] differential in the *Chlamydia* lifecycle, suggestive of a bacterial host-stress sensing mechanism. Using photobleaching approaches, we established that the RB is the master regulator of this [K^+^] differential. We also observe a fast K^+^ exchange between RBs revealing a role for this ion in inter-bacterial communication. Finally, we experimentally demonstrate the importance of spatio-temporal parameters, here [K^+^] associated with membrane potential, in the RB to EB differentiation process. Together, our data reveal that *Chlamydiae* use K^+^ electrical signalling to sense the host cell, ensure community communication and to orchestrate critical steps in the *Chlamydia* lifecycle.

## 2. Experimental Procedures

### 2.1. Cell Culture and Infection

HeLa human cervical epithelial cells and RL95-2 uterine endometrial cells were cultured as recommended by American Type Culture Collection.

*Chlamydia trachomatis* serovars LGV2 and D and *Chlamydia muridarum* were routinely propagated in HeLa cells as previously described [22].

*C. trachomatis* serovar LGV2 has been transformed with an empty pASK-GFP-L2 as described [23].

### 2.2. Drug Treatments

Nigericin, valinomycin, chloride ionophore I, ionomycin, glibenclamide, and doxycycline were purchased from Sigma. They were titrated to concentrations that could be tolerated by ≥75% of cells. All ionophores and doxycycline were used at 1 µM, and glibenclamide at 10 µM. At appropriate time points, *Chlamydia*-infected cells were treated. Drugs were continuously incubated with cultured cells except during recovery experiments in which they were removed by repeated washing with phosphate buffered saline (PBS) and the replacement of the culture media at the appropriate time. Controls contained the corresponding volume of the solvent used to dissolve the active molecule.

### 2.3. Persistence Induction

For adenosine/erythro-9-(2-hydroxy-3-nonyl)adenine (Ad/EHNA) (Sigma, St Louis, MO, USA) induction, infected cells were treated as previously described [24]. For IFNγ-induced persistence, 20 U/mL of IFNγ (Sigma) were added at 12 hpi (hours post infection). In both cases, cells were fixed or prepared for live imaging at 24 hpi.

### 2.4. Flame Photometry

HeLa cells were seeded at 70% confluence in 6-well tissue culture plates and treated for 6h with the indicated ionophore. Cells were lysed with 0.5% (*v*/*v*) Triton X-100 in deionised water on ice for 30 min followed by centrifugation at 8000× *g*. Samples were then siphoned into the flame photometer and emission intensity at the specific K^+^ emission wavelength (766 nm) was recorded [25]. Emission intensity was converted into [K^+^] using a calibration curve obtained in parallel using a K^+^ dilution series in deionised water (Figure S1A), and a ratio calculated [K^+^]_x_/[K^+^]_control_ (x being the sample).

### 2.5. Fluorescence Labelling

Infected cells were fixed in phosphate buffered saline (PBS) containing 4% buffered paraformaldehyde (*w*/*v*) at appropriate time points. Mitochondrial labelling was performed as previously described [22]. Cells were then permeabilised in methanol/ethanol (*v*/*v*) (5 min, 4 °C) prior to labelling. DNA labelling was performed using DRAQ-5 DNA (Abcam, Cambridge, UK). FITC conjugated-anti-*Chlamydia* MOMP/LPS (Argene, Biomerieux, Marcy-l’Etoile, France) was incubated 1 h in PBS-1% (*w*/*v*) BSA.

For live experiments, HeLa cells were seeded at 70% confluence in LabTek live microscopy chambers (Nunc) and treated as required. Cells were labelled using 2 mM asante potassium green 2 (APG-2), 5 µM DRAQ5 and/or 5 nM of Mitotracker red diluted in DMEM without phenol red (45 min, 37 °C, 5% CO_2_). Media containing probes but no phenol red was renewed and cells were imaged using a confocal microscope.

### 2.6. Confocal Microscopy

Samples were observed using a confocal microscope (TCS Sp5 AOBS; Leica, Wetzlar, Germany), using settings as described [22].

For live experiments of non-infected cells, or following treatment with nigericin or valinomycin recording started immediately after addition of the ionophore or control solvent, and the APG-2 channel was recorded for 40 min, every 5 min.

For live experiments of cells infected with *C. trachomatis* LGV2, parameters were set to image a scan every 1.509 s.

For photobleaching experiments, the Leica FRAP wizard software was used with 20 frames pre-bleached/10 frames bleached/60 frames post-bleached sequence. Bleaching was performed on user-prescribed regions of interest (ROI), using all argon laser lines set at 100%. A scan was imaged every 1.509 s.

### 2.7. Image Analysis

All images were analyzed using Fiji [26] or ImageJ [27]. For all analyses, number of events are greater than 30.

To measure the fluorescence intensity of different compartments, the average background intensity measured from an area with no cells was subtracted from the corresponding ROIs. Class averages of intensity were calculated manually by grouping similar intensities into the indicated classes.

To quantify mKate expression, APG-2 intensity or Mitotracker accumulation with respect to bacterial diameter, automated quantification was performed (Figure S1B). Segmentation of the bacteria was conducted as described [22]. Each individual particle was associated with a number. This allows (1) the exclusion of particles which are not bacteria, as well as groups of associated bacteria; (2) the measurement of the diameter; and (3) the localisation of the bacteria in the inclusion. The resulting mask from the DRAQ-5 channel was then used to measure the mean intensity of mKate or APG-2.

For photobleaching experiments, to correct for background and internal photobleaching, whole cells were imaged for the equivalent time (90 frames at 1.509 s per frame). Using a macro, the mean intensity of fluorescence of a user-designed ROI was measured along the t-axis. This allows the calculation of I_F1_/I_Ft_ were I is the mean intensity, F: frame, and t: time (in frame). This allows the calculation of the correction factor ‘B’ for each frame. Consequently, during the analysis of the photobleaching experiments, the following formula was used to correct for internal variation, according to Kappel and Eils [28]: I_corrected_ = (I_t_ -− I_background_) * B_t_. All data are the average of four independent experiments where multiple ROIs from RBs, cytosol and inclusion lumen from different cells and backgrounds were analysed.

### 2.8. Infectivity Assay

Titration was performed as described [22].

### 2.9. rRNA Isolation and Amplification

At 50 hpi, HeLa cells were collected and total RNA isolated and purified as previously described [29]. Primers were designed for bacterial 16S RNA -FW: 5′-CGCCTGAGGAGTACACTCGC- and -RV: 5′-CCAACACCTCACGGCACGAC- and eukaryotic 18S RNA -FW: 5′-ATGGCCGTTCTTAGTTGGTG and -RV: 5′-CGCTGAGCCAGTCAGTCAGTGTAG. Control PCR reactions using bacterial 16S primers were carried out on isolated RNA to verify the absence of genomic DNA.

### 2.10. Transmission Electron Microscopy (TEM)

HeLa cells were seeded, infected and treated as previously described. At 24 hpi, cells were fixed in 0.1 M sodium cacodylate trihydrate containing 2.5% gluteraldehyde (*w*/*v*) and 0.05% malachite green (*w*/*v*) (2 h, room temperature). Cells were then stained using 0.5% osmium tetroxide (*w*/*v*) (30 min, room temperature) followed by 1% uranyl acetate (*w*/*v*) (1 h, room temperature) prior to dehydration using ethanol and embedding in agar 100 resin (Agar Scientific, Stansted, UK). Resultant blocks were then sectioned onto 400 mesh copper grids and observed using a Tecnai 100 (FEI, Lausanne, Switzerland).

## 3. Results

### 3.1. Using APG-2 as a Potassium Ion (K ^+^) Probe for Live Cell Imaging

**Figure 1:**
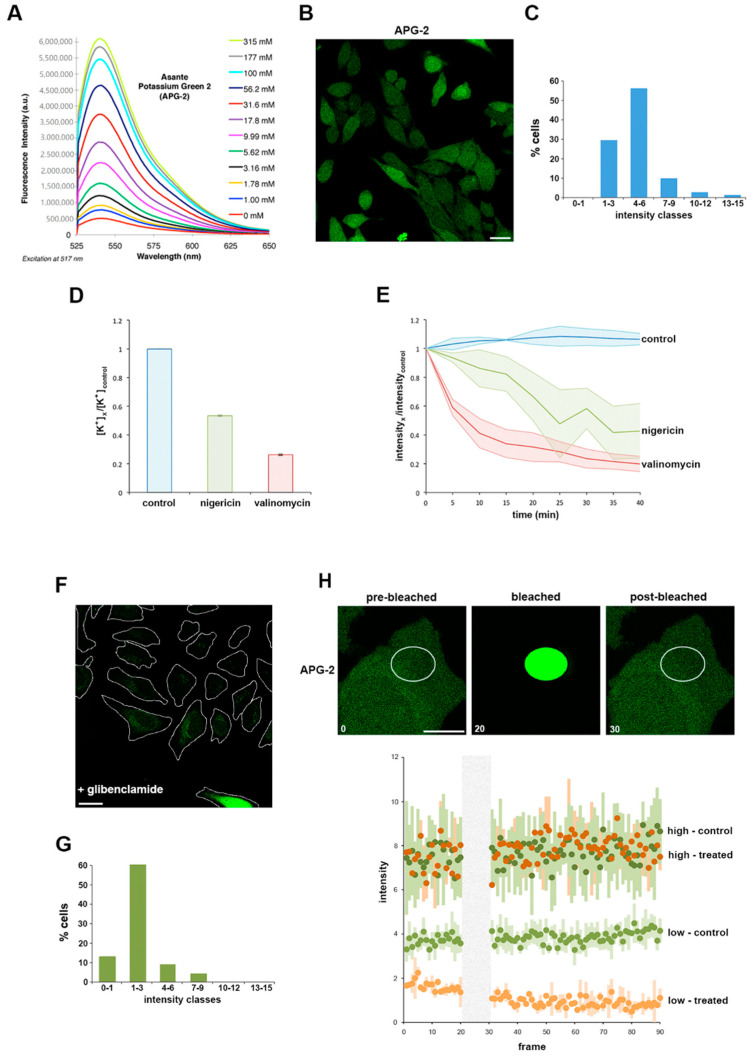
Asante potassium green 2 (APG-2), a valid tool to study K^+^ by live cell imaging. (**A**) Provided by supplier (with permission), variation of APG-2 intensity depending on the K^+^ concentration (different coloured lines). (**B**) HeLa cells were labelled with APG-2 and imaged using confocal microscopy. Scale bar: 25 µm (**C**) Measured average cytoplasmic APG-2 intensity of non-infected non-treated cells were classified according the *x*-axis. (**D**) HeLa cells were treated with nigericin (green), valinomycin (red) or untreated (blue). Following this, 6 h later, [K^+^] was determined flame photometry as indicated (Materials and Methods and Figure S1A) and normalised to the control. (**E**) HeLa cells were labelled with APG-2 and imaged using confocal microscope every 5 min for 40 min. (**F**) HeLa cells were treated with glibenclamide 12 h prior to labelling with APG-2 followed by confocal imaging. Scale bar: 25 µm. (**G**) Class average intensity of APG-2 performed as in (**C**) in glibenclamide treated cells as represented in (**F**). (**H**) Non-treated HeLa cells as in (**B**) or HeLa cells treated with glibenclamide as in (**F**) were subjected to photobleaching of a portion of their cytoplasm as presented in the upper panels. Analysis of the APG-2 fluorescence intensity (lower panel) over time was performed for the different conditions: control cells are represented in two shades of green (dark: high intensity or bright: low intensity) and glibenclamide-treated cells are represented by two shades of orange (dark: high intensity or bright: low intensity). High intensity: from categories 4–6 and beyond as in (**C**) and (**G**). Low intensity: categories 0–1 and 1–3 as in (**C**) and (**G**).

Assante potassium green 2 (APG-2) is a K^+^ chelator coupled via a phenyl linker to a fluorophore. When measured by fluorimetry in solution, the fluorescence intensity is directly proportional to the concentration of potassium ions [K^+^] (Figure 1A). To initially establish whether APG-2 could analogously be employed to quantify [K^+^] in cultured mammalian cells, HeLa cells were incubated with APG-2 and imaged by confocal microscopy. Cells co-incubated with the probe exhibited diffuse labelling throughout the cytoplasm (Figure 1B), confirming the ability of the probe to penetrate the cells and report the 20-fold enrichment in intracellular [K^+^] compared to the surrounding extracellular medium. Furthermore, classification of cells based on their average cytosolic APG-2 signal intensity shows a bell-shaped distribution (Figure 1C), consistent with the expected variation in cytoplasmic [K^+^] [30]. However, the low classes (1 to 6) represent the majority of the cells (90%). These data show that APG-2 intensity reflects [K^+^]_cytosol_ in cultured cells.

**Figure 2:**
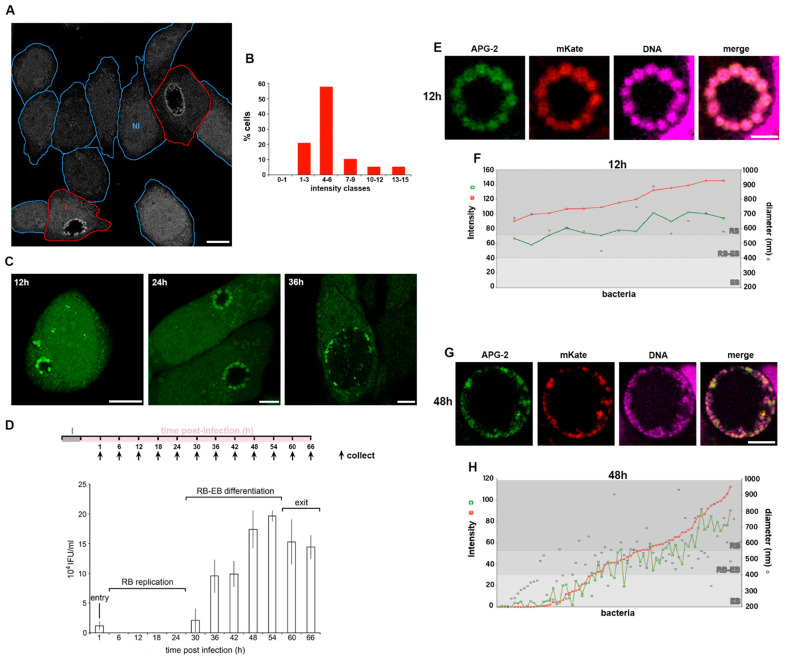
*C. trachomatis* RBs accumulate K^+^. HeLa cells were infected with *C. trachomatis* LGV2. (**A**) Infected cells were incubated with APG-2 24 hpi and a mixed population of infected and non-infected cells imaged. Cell edges are indicated in blue for non-infected cells (NI) and in red for infected cells (I). Scale bar: 10 µm. (**B**) Measured average cytoplasmic APG-2 intensity of non-infected cells were classified according the *x*-axis. (**C**) Infected cells at 12, 24 and 36 hours post infection (hpi) were incubated with APG-2 and imaged. Scale bar: 10 µm. (**D**) Upper panel: schematic of the experiment design. I: infection. Lower panel: Infectivity assay of cells collected at the indicated time on the *x*-axis corresponding to the collection time on the upper panel. The *y*-axis shows the inclusion forming unit per mL (IFU/mL). The different phases of infection corresponding to the infectivity assay are indicated. Error bars: standard deviation. (**E**–**H**) cells were infected with *C. trachomatis* LGV2 as in (**A**) to (**D**), but bacteria were transformed with pASK-GFP-L2 allowing the expression of mKate (red) only when the bacteria are in the RB form. Cells were incubated with APG-2 at the indicated time point ((**E**,**F**) 12 hpi; (**G**,**H**) 48 hpi) and imaged. Image analyses were performed as described (Experimental Procedures and Figure S1B) allowing the measurement of the bacterial diameter, the intensity of APG-2 (green) and mKate (red) for each individual bacteria ((**F**) and (**H**), grey level reflecting different type form of the bacteria).

To verify that the apparent variation in APG-2 fluorescence intensity specifically reports cellular [K^+^], cultured HeLa cells were next treated with two well-characterised potassium ionophores, nigericin and valinomycin, which artificially induce K^+^ efflux through their actions as a K^+^/H^+^ antiporter and a K^+^ uniporter, respectively [31]. Flame photometry was initially used to directly quantify the effect of these ionophores on intracellular [K^+^]. Both valinomycin and nigericin induced a significant decrease in intracellular [K^+^], with valinomycin having a greater effect under our specific experimental conditions (Figure 1D and Figure S1A; see also Experimental Procedures). Valinomycin or nigericin ionophores were then added to cultured HeLa cells pre-labelled with APG-2 (i.e., as shown in Figure 2B). Fluorescence intensity analysis over time showed ionophore-induced intensity decreases mirroring by fold to those determined by flame photometry that reached steady-state after ~35 min (Figure 1D,E). These data validate that APG-2 accurately reports intracellular [K^+^] in live cultured cells, and that [K^+^] can be monitored over time using fluorescence imaging techniques.

To establish the extent to which it is possible to employ APG-2 to monitor [K^+^] flux in cells, we exploited glibenclamide, an inhibitor of inward rectifying K^+^ channels coupled to photobleaching and fluorescence recovery assays. Glibenclamide reduces K_ir_ activity by 50–60% [32], attenuating the ability of a cell to rectify its intracellular [K^+^], therefore glibenclamide treatment is partial and only affect cells actively rectifying their intracellular [K^+^] using this type of channels [33]. Consistent with this, when APG-2 labelled HeLa cells were treated with glibenclamide, cells were unable to replenish the cytoplasm with K^+^, as reported by a significant reduction in APG-2 fluorescence intensity monitored by microscopy (Figure 1F). When glibenclamide-treated cells within this population were classified on the basis of their average APG-2 signal intensity a bell-shaped distribution was again evident, but with the maxima shifted towards the lower intensity classes when compared to the untreated cell population (Figure 1C,G). Based on these data, we reasoned that the cells with a lower APG-2 intensity (classes 0–1 and 1–3 in Figure 1G) replenish their depleted cytoplasmic [K^+^] by using inward rectifying channels, while the population exhibiting a higher APG-2 fluorescence intensity (classes 4–6 onwards in Figure 1G) must have reached stability. To examine this further, we employed fluorescence recovery after photobleaching assays to investigate [K^+^] flux in these two cell populations in more detail. APG-2 fluorescence was photobleached within a specific cytoplasmic region of interest, and the kinetics of recovery monitored over time (Figure 1H). As predicted, cells exhibiting high initial APG-2 fluorescence intensity remained unaffected by the glibenclamide treatment, as the intensity profile in both non-treated and treated cells was similar after photobleaching and recovery (Figure 1H). By comparison, the cells with lower initial APG-2 fluorescence intensity showed a reduced ability to recover following glibenclamide treatment demonstrated by a difference between the non-treated and treated cell profiles (Figure 1H). These data show that it is possible to track [K^+^] in live cells using confocal microscopy.

### 3.2. Potassium Ions Accumulate within Replicative C. trachomatis Reticulate Bodies

To monitor the distribution of K^+^ ions in *Chlamydia* infected cells, HeLa cells were infected with *C. trachomatis* LGV2 and labelled using APG-2 at 24 hpi (hours post infection) (Figure 2A). As previously demonstrated, non-infected HeLa cells exhibit a variation in cytoplasmic [K^+^] (Figure 1B,C). When assayed under equivalent conditions, cells infected by *C. trachomatis* show an identical distribution of host cytoplasmic [K^+^] (Figure 2A,B), showing that bacterial infection does not significantly change cytoplasmic [K^+^]. However, in infected cells, bacteria within the inclusion strongly accumulate APG-2 (Figure 2A). When HeLa cells infected with *C. trachomatis* LGV2 were visualised during the infection cycle between 12 hpi and 36 hpi differences in the distribution of APG-2 within the inclusion were evident (Figure 2C). At 12 hpi and 24 hpi multiple distinct bright structures approximately 1 µm in diameter are present at the periphery of the inclusion (Figure 2C 12 hpi and 24 hpi), which have significantly reduced in number by 36 hpi (Figure 2C 36 hpi). Infectious non-replicative elementary bodies (EBs) and non-infectious replicative reticulate bodies (RBs) are characterised by differences in their diameter at ~300 nm and ~1 µm, respectively [34] and by their location within the lumen of the inclusion. Most RBs remain in close proximity to the luminal face of the inclusion membrane to enable communication with the host cell, whereas EBs disengage and move into the lumen [35]. These observations imply that RBs accumulate more K^+^ than EBs, which potentially equilibrate their [K^+^] with the inclusion lumen.

To confirm that the APG-2 (K^+^) enrichment happened within the RB, we defined the different stages of the *C. trachomatis* LGV2 lifecycle in HeLa cells under our conditions. An infectivity assay was used to monitor infectious EBs from 0 hpi (cell entry) to 66 hpi (cell exit) (Figure 2D). These data show the characteristic phases of entry (low infectivity from entering EBs), RB replication (no infectivity), RB-EB differentiation (increasing infectivity) and exit (plateau or reduced infectivity), demonstrating that *C. trachomatis* LGV2 is predominantly in the non-infectious RB form from 6 hpi to 24 hpi (Figure 2D). This correlates with time points where APG-2 accumulation occurs proximal to the inclusion membrane, strongly reinforcing the view that replicating *C. trachomatis* RBs accumulate K^+^. Finally, we adopted a segmentation approach using confocal microscopy in combination with genetic manipulation to precisely localise EBs and RBs. HeLa cells were infected with *C. trachomatis* LGV2 transformed with a plasmid that expressed mKate only in RBs [23] and their diameter was assessed using a nucleic acid probe (DRAQ-5) (Figure S1B) simultaneously with APG-2 labelling (Figure 2E,F). Since DRAQ-5 labelled both EBs and RBs, this confirmed that both developmental forms of the bacteria remain accessible to small probes such as APG-2, thus excluding the hypothesis that EB could be impermeant. At 12 hpi, the inclusion contains a single population of bacteria expressing mKate enriched with APG-2 (Figure 2E), with a diameter of 500–1000 nm characteristic of RBs (Figure 2F). By 36 hpi, the inclusion contains a heterogenous population of bacteria (Figure 2G), with diameters corresponding to EBs (200–400 nm), RBs (550–1000 nm) and intermediate forms (400–550 nm) (Figure 2H). mKate intensity increases proportionately with bacterial diameter (Figure 2F,H) consistent with expression within RBs [23]. Strikingly, APG-2 and mKate intensity increase correlatively (R = 0.957) (Figure 2F,H and Figure S1C), and concomitantly with bacterial diameter (Figure 2H), clearly demonstrating that the *C. trachomatis* RBs specifically accumulate K^+^.

### 3.3. The Role of K^+^ in the Bacterial Stress Response

**Figure 3:**
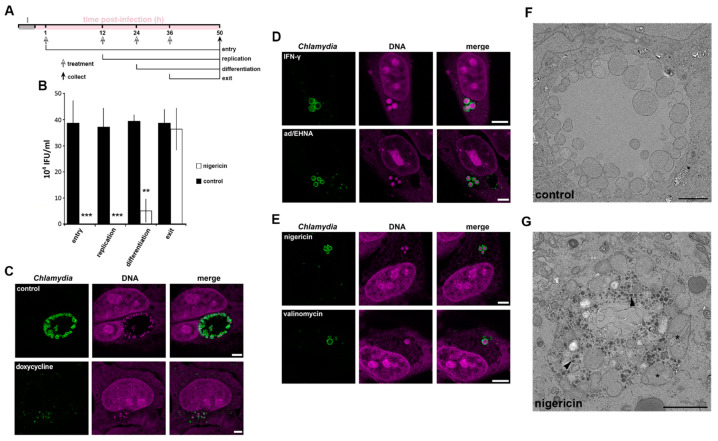
Effect of K^+^ ionophores on chlamydial infectivity and morphology. HeLa cells were infected with *C. trachomatis* LGV2. (**A**) Schematic of the experiment design associated with panel B. I: infection. Time of treatment, corresponding to the different phases of the *Chlamydia* lifecycle as indicated, and collection have been determined based on the titration assay (Figure 2D). (**B**) Infected cells were treated with nigericin (white bars) or untreated (black bars) at the indicated phase of the lifecycle (*x*-axis) and samples collected at 50 hpi. Infectivity was assessed and IFU/mL indicated on the *y*-axis. ** *p* < 0.01, *** *p* < 0.005. Error bars: standard deviation. For (**C**–**E**) cells were treated as indicated at 12 hpi and fixed at 24 hpi prior to labelling with anti-*Chlamydia* (green) and DNA probe (magenta). Scale bar: 5 µm. (**C**) upper panels: non-treated, lower panels: doxycycline (**D**) upper panels: IFN-γ lower panels: adenosine (Ad/EHNA) (**E**) upper panels: nigericin, lower panels: valinomycin. (**F**,**G**) control infected cells (**F**) or treated at 12 hpi with nigericin (**G**) prior to processing for transmission electron microscopy at 24 hpi. Scale bar: 2 µm.

Given the specific enrichment of K^+^ within *C. trachomatis* RBs, we next explored the potential importance of this ion during the bacterial infection cycle by artificially interfering with the cytoplasmic [K^+^] in the host cell. HeLa cells were infected with *C. trachomatis* LGV2 and treated with the K^+^ ionophore nigericin at the specific phases of the infection cycle previously identified (Figure 2D and Figure 3A). Following ionophore addition, the cycle was allowed to progress to 50 hpi when bacterial infectivity was determined and compared to controls (Figure 3B). In comparison to controls in which infectivity remained constant (Figure 3B, black bars) a significant decrease in infectious progeny was evident when infected cells were treated with nigericin prior to RB-EB differentiation. Indeed, during this stage the majority of bacteria are RBs, while treatment after differentiation, when the majority of the bacterial population are EBs had no effect on the recovery of infectious progeny (Figure 3B). These data suggested that RBs, which would specifically accumulate K^+^ (Figure 2), are either killed or that stressed RBs enter a persistent form upon the addition of the K^+^ ionophore nigericin. Finally, we assessed the specificity of the role of K+ in the *C. trachomatis* LGV2 life cycle, infected HeLa cells were treated with various ionophores. An ionophore specific for sodium ions (Na^+^) over K^+^ is yet to be described [36], however ionophores which specifically disrupt calcium (Ca^2+^; ionomycin) or chloride (Cl^−^; chloride ionophore I) had no significant effect on bacterial infectivity in equivalent assays (Figure S2A). Moreover, a similar dependency for K^+^ during RB replication was evident when uterine RL95-2 epithelial cells were infected with *C. trachomatis* LGV2, HeLa cells infected with urogenital *C. trachomatis* serovar D and when HeLa cells were infected with *C. muridarum* (Figure S2B–H). Taken together these findings reveal that K^+^ ions are specifically required for the development of the RB form of *Chlamydia*, an effect independent of host cell type and bacterial strain and species. Disruption of the [K^+^] in the host cytoplasm induces a loss of infectivity, which could be attributed to either the death of the RB or a shift into a persistent form.

One method to distinguish death and persistence is to examine the behaviour and morphology of the bacteria in the infected host cell [29,37]. To assess whether K^+^ restriction in the host cytoplasm was phenotypically reminiscent of chlamydial death or persistence, we compared bacterial and inclusion morphology in HeLa cells infected with *C. trachomatis* LGV2 following treatment with an antibiotic known to kill *Chlamydiae* (doxycycline), two known inducers of persistence (IFN-γ or adenosine/EHNA) or K^+^ ionophores (nigericin and valinomycin) [24]. Doxycycline treatment rendered the bacteria unable to form an inclusion, which appeared compromised and disrupted (Figure 3C). By contrast, IFN-γ or adenosine/EHNA (ad/EHNA) treatment induced the formation of distinct and discretely clustered persistent bodies (PBs), which are characterised by an increased diameter (2 µm ± 0.45) (Figure 3D). Strikingly similar aberrant bacterial forms reminiscent of persistence were induced following treatment with the K^+^ ionophores nigericin or valinomycin (Figure 3E), whereas equivalent treatment with Ca^2+^ or Cl^−^ ionophores did not affect bacterial or inclusion morphology (Figure S2A,I). Transmission electron microscopy (TEM) of infected cell sections was used to observe the aberrant bacterial forms resulting from K^+^ ionophore treatment and the structure of the inclusion in greater detail (Figure 3F,G). The TEM data revealed that the inclusions present in mock (Figure 3F) or K^+^ ionophore-treated (Figure 3G) HeLa cells maintain their integrity, possessing an intact inclusion membrane and a visible distinction in density between the inclusion lumen and the host cell cytosol, showing that ionophore treatment does not directly rupture or grossly disrupt the structure of the inclusion membrane. The bacteria within the inclusion remain individually distinct following K^+^ ionophore treatment, yet specifically adopt an aberrant morphology (Figure 3G). These bodies morphologically resemble previously described PBs [2]. Additionally, electron dense material accumulates specifically within the lumen of the inclusions following K^+^-ionophore treatment consistent with the accumulation of glycogen observed during *C. trachomatis* persistence [38]. Taken together, these data suggest that disruption of the [K^+^] in the host cell cytoplasm leads to chlamydial persistence and the conversion of RBs into PBs, rather than to bacterial death.

**Figure 4:**
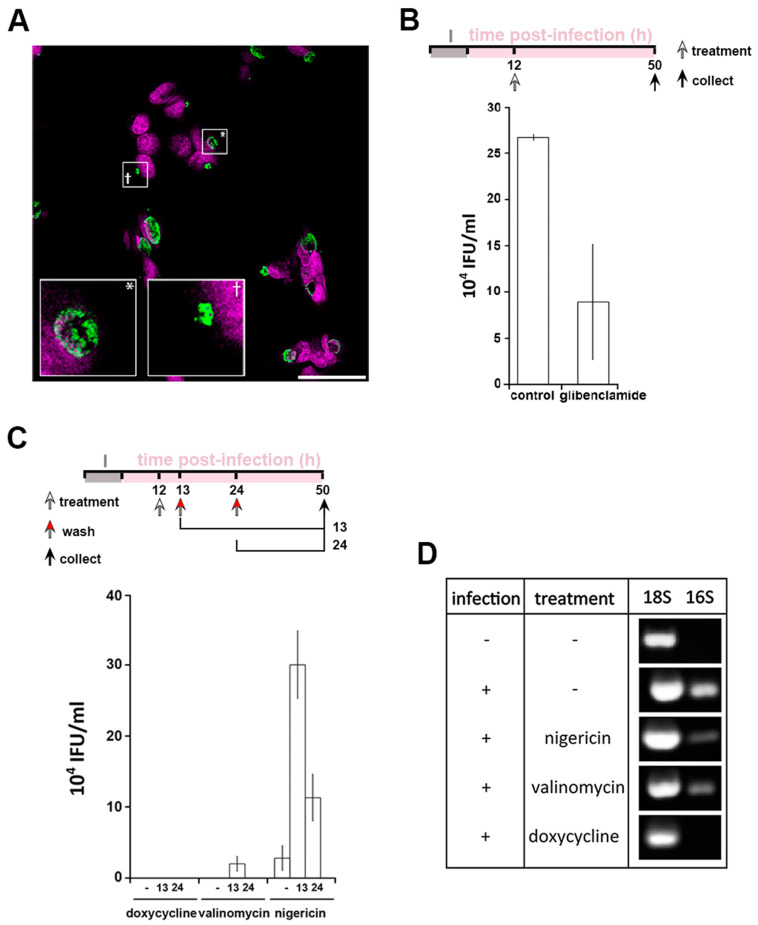
Interfering with cytoplasmic [K^+^] leads to *Chlamydia* persistence. HeLa cells were infected with *C. trachomatis* LGV2. (**A**) Cells were treated with glibenclamide 12 hpi prior to fixation at 24 hpi followed by labelling with anti-*Chlamydia* (green) and DNA probe (DRAQ-5, magenta). Insert shows a normal inclusion (*) and abnormal inclusion (+) at higher magnification. Scale bar: 25 µm. (**B**) upper panel: schematic of the experiment design. I: infection. Lower panel: after treatment with glibenclamide and sample collection as described in the upper panel, lysate were tested for infectivity by measuring inclusion forming units per mL (IFU/mL). (**C**) Upper panel: schematic of the experiment design. I: infection. Lower panel: reversion of persistence assay, performed as described in the upper panel. Infectivity assays were performed (IFU/mL: inclusion forming unit per mL). For (**B**) and (**C**), error bars: standard deviation. (**D**) At 12 hpi, cells were treated with 1 µM nigericin, valinomycin or doxycycline. At 50 hpi, cells were prepared for RNA expression analysis by RT-PCR using 18S as a loading control and 16S as a marker of bacterial viability.

Persistence is a mechanism which allows *Chlamydia* to survive under conditions of environmental stress. However, the effects of ionophores can be pleiotropic, as for example both nigericin and valinomycin induce mitochondrial stress [31] (Figure S3A). Therefore, independent modification of intracellular K^+^ was performed using glibenclamide, an inhibitor of a subset of potassium channels (Figure 1F–H). In this scenario, the replenishment of the cytoplasmic [K^+^] is disrupted leading to a milder modification of the [K^+^]_cytoplasm_ but has the advantage of avoiding the mitochondrial stress induced by ionophore treatment (Figure S3A). Treatment with glibenclamide at 12 hpi induced the formation of PBs in 29% ± 1.7% of the infected cells (Figure 4A). However, a reduction in infectivity by approximately 60% is observed following glibenclamide treatment when compared to an equivalent control infection (Figure 4B). This discrepancy between bacterial morphology and infectivity suggests a more subtle relationship between the bacteria and intracellular K^+^. In agreement with this, glibenclamide treatment of different cell types infected with different species and serovars of *Chlamydia* also significantly reduced bacterial infectivity in every combination (Figure S3B–D). Together, these data demonstrate that PBs are formed in response to reduced [K^+^]_cytoplasm_.

A defining feature of persistence is the ability of the bacteria to recover after the removal of a persistence stimulus. We first established that PBs form as soon as 30 min after K^+^ ionophore treatment (Figure S3E). Therefore, to investigate the effect of removing the stimuli, infected cells were treated at 12 hpi for 1 or 12 h, prior to washing at 13 hpi or 24 hpi, respectively. Doxycycline and nigericin are reversible agents whereas valinomycin irreversibly intercalates into biological membranes [39] making it unlikely to be washed out. Quantification of bacterial infectivity showed that doxycycline treatment leads to bacterial death (Figure 4C). As expected, the recovery of infectivity was meagre in the presence of valinomycin but nevertheless present after 1h treatment only, infectivity likely resulting from a residual population of EBs unaffected by the treatment. Interestingly, after nigericin treatment and subsequent removal, infectivity can be detected after both 1 h and 12 h treatments (Figure 4C). This demonstrates that the PBs formed by K^+^ depravation can be defined as canonical persistent bodies. The viability of PBs induced by K^+^ ionophores was assessed by measuring the expression of bacterial 16S rRNA [40] in comparison to eukaryotic 18S rRNA (Figure 4D). Bacterial 16S rRNA was not detected following doxycycline treatment while it is present following both nigericin and valinomycin treatment demonstrating that the bacteria remain viable under the latter conditions (Figure 4D). Taken together these data demonstrate that cytoplasmic [K]^+^ reduction induces a bacterial stress response, whereby *Chlamydia* enter a persistent state.

### 3.4. Disruption of [K^+^] Is a Common Aspect of Chlamydia Persistence

**Figure 5:**
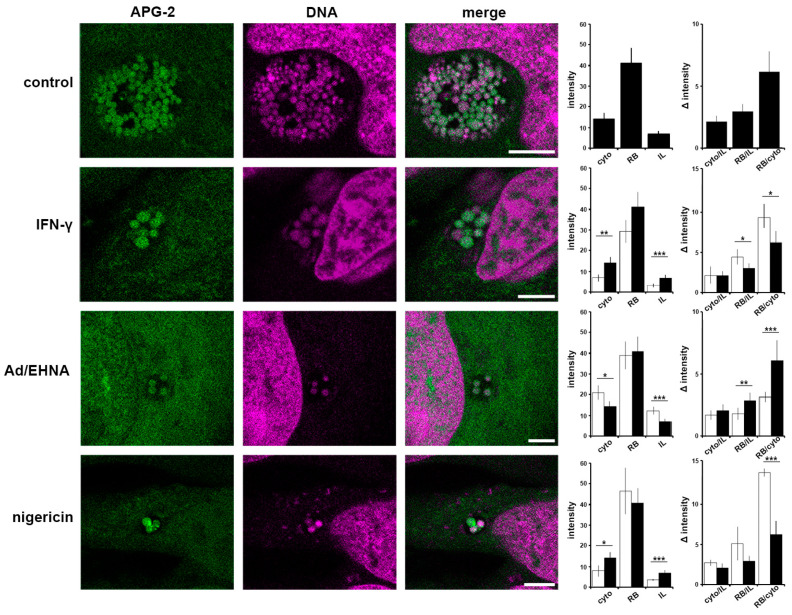
Critical importance of the interface between the bacteria and host cytosol. HeLa cells were infected with *C. trachomatis* LGV2. At 12 hpi cells were treated IFN-γ, Ad/EHNA (adenosine) or nigericin and incubated for a further 12 h. At 24 hpi, cells were incubated with APG-2 and DNA probe (DRAQ-5, magenta) and imaged using confocal microscopy (**left panels**). Scale bars: 5 µm. Image analyses allowed the comparison of the average APG-2 intensity in the cytoplasm (cyto), reticulate bodies (RB) and inclusion lumen (IL), and a ratio determined (**right panels**). Black column: non-treated, white column: treated. Error bars: standard deviation. * *p* < 0.05, ** *p* < 0.01, *** *p* < 0.005.

We demonstrated that interfering with the [K^+^]_cytoplasm_ induces persistence following treatment with potassium ionophores or glibenclamide. We next investigated [K^+^] distribution during nigericin-induced persistence, as well as the more physiologically relevant IFN-γ- or adenosine-induced persistence (Figure 5). It has been demonstrated that IFN-γ inhibits the Na^+^/K^+^ ATPase pump, which will induce a decrease in cytoplasmic [K^+^] [41]. In contrast, adenosine activates a specific population of inward rectifier K^+^ channels [42,43]. Comparing both treatments will therefore allow the impact of a reduction or an increase in the intracellular [K^+^] to be monitored. In control cells at 24 hpi RBs exhibit the highest intensity, followed by the cytoplasm (cyto), in which APG2 is approximately half the intensity, and the inclusion lumen (IL), which demonstrates that the lumen is not a passive compartment simply equilibrating with the cytoplasm but reflects a controlled [K^+^] environment. As expected, treatments with IFN-γ, Ad/EHNA or nigericin- induce changes in [K^+^]_cytoplasm_ (Figure 5). Interestingly, in each of these scenarios, and irrespective of the induced variation in the [K^+^]_cytoplasm_, the fluorescence intensity of the bacteria is not modified, demonstrating that PBs retain the ability to control their [K^+^]. Additionally, the IL intensity remains lower than the RBs and the cytosol, confirming that these different stressors do not disrupt the integrity of the inclusion. The fluorescence intensity ratio between the host cytoplasm and the inclusion lumen (cyto/IL) remains similar when the infected cells are exposed to different stressors, suggesting a controlled exchange occurs between the two compartments. However, the RB/cyto ratio is most significantly affected by the different treatments, demonstrating that the RBs can sense stress in the host cell via the interface between the RB, the inclusion membrane and the host cytosol, already implicated in effector delivery [22].

Together, these data demonstrate that the RB, IL and cytosol are three compartments with distinct [K^+^], and that stressors modify the ratio between the RB-cytosol and RB-IL suggesting a critical role for these RB interfaces.

### 3.5. A Role for K^+^ in Host Pathogen Communication

**Figure 6:**
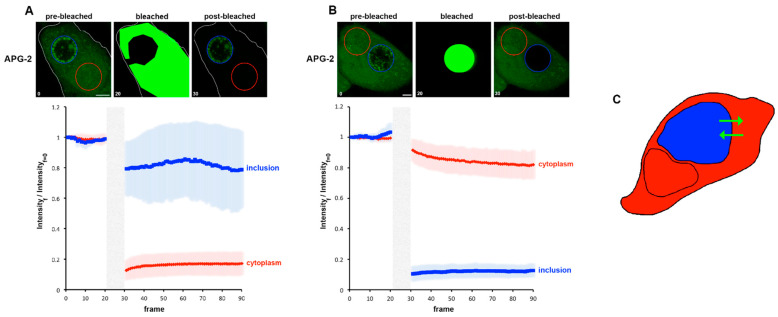
Fast movement of K^+^ between the host cytoplasm and the inclusion. HeLa cells were infected with *C. trachomatis* LGV2. At 24 hpi cells were labelled with APG-2 and then imaged. APG-2 was bleached in the cytoplasm (**A**) or the inclusion (**B**), and imaged again using confocal microscopy, as represented in the upper panel where one cell is shown as a representative example. Circles represent regions of interest: inclusion (blue) and cytoplasm (red). Scale bar: 5 µm. On the lower panel, analysis of fluorescence intensities of the inclusion of cytoplasm are represented related to the frame number (a frame per 1.509 s). The light grey boxes represent the bleaching step. (**C**) Schematic of the result, the cell is depicted, and the cytoplasm is coloured in red while the inclusion is in blue. The green arrows represent the proposed K^+^ movement.

We next extended these approaches to investigate K^+^ flux in infected cells. Photobleaching experiments, as described in Figure 1H were performed using *C. trachomatis* infected cells labelled at 24 hpi to measure the possible recovery of fluorescence of the bleached area as well as the loss in fluorescence in unbleached regions. Due to the inherent Brownian motion of the bacteria, only short post-bleached time courses could be recorded. In parallel, DRAQ-5 was used to define the RBs and IL. First, we simply considered the cytoplasm and the entire inclusion. Each compartment was bleached in turn and the resulting behaviour of cytoplasm and the inclusion recorded (Figure 6A,B). Each bleached compartment exhibits a clear post-bleach decrease in the APG-2 intensity, and it is also clear that the counterpart compartment is also affected. This demonstrates that the two compartments are actively exchanging potassium (Figure 6C).

**Figure 7:**
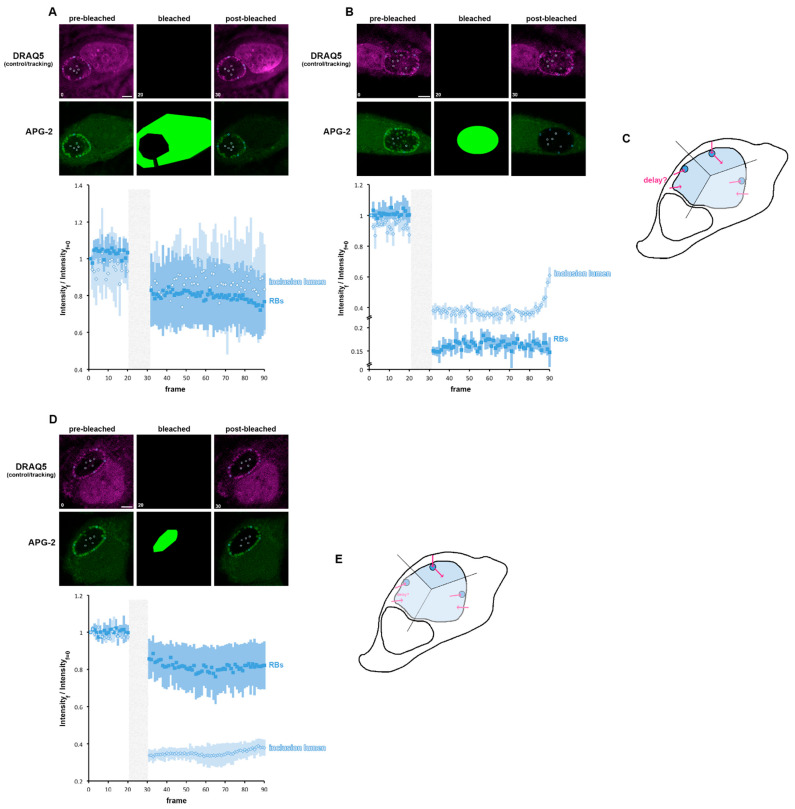
K^+^ flux is controlled by the reticulate body. (**A**,**B**) HeLa cells were infected with *C. trachomatis* LGV2. At 24 hpi cells were labelled with APG-2 and DRAQ-5 and then imaged. APG-2 was photobleached as indicated on the upper panels in the cytoplasm (**A**) or in the whole inclusion (**B**). Circles show the regions of interest used for the analysis of the intensity depending on the frame (lower panels: 1.509 s/frame) presented on the lower panels. Dark blue: reticulate bodies (RBs) and light blue for the inclusion lumen. Scale bar: 5 µm. (**C**) schematics showing the proposed K^+^ flux (pink arrows) between the host cytoplasm (white) the inclusion lumen (light blue) and the bacteria (dark blue). (**D**) HeLa cells were infected with *C. trachomatis* LGV2. At 24 hpi cells were labelled with APG-2 and DRAQ-5 and then imaged. APG-2 was photobleached in the inclusion lumen. (**E**) schematics showing the proposed K^+^ flux (pink arrows) between the host cytoplasm (white) the inclusion lumen (light blue) and the bacteria (dark blue).

The inclusion is composed of the IL and the RB population. Consequently, we next observed the consequences of bleaching either the cytoplasm or the entire inclusion, on the behaviour of the IL or RB (Figure 7A,B). When the cytoplasm is bleached, RBs and IL were equivalently affected, with a clear post-bleached decay. A slight difference in the trend of both compartments could be observed especially towards the end of the recorded time course, suggesting a subtle difference in the K^+^ flux between the two compartments (Figure 7A). This is clearest when the cytosol has been bleached (Figure 7B). Indeed, as expected, both the RB and IL have been affected: the RBs partially recovered quickly after bleaching and reach a steady state, whereas the IL does not initially replenish after bleaching but does so abruptly towards the end of the recording time course. This confirms that the K^+^ flux is clearly different between the IL and the RB. This allowed us to propose three possible scenarios: (i) that K^+^ initially flow into the IL and then are pumped in the RB, a possibility not supported by our data (Figure 7B), (ii) that K^+^ are initially pumped into the RB and could then reach the IL or (iii) that K^+^ initially flow from the cytosol into the IL or RB independently, with cytoplasm to IL transport being slower than the cytoplasm to RB pathways. To decipher between scenarios (ii) and (iii), we bleached the IL and observed the behaviour of the RBs and the IL (Figure 7D). The IL is affected immediately after bleaching, as expected, but does not begin to recover until later in the time course. Conversely, the RBs are affected immediately after bleaching and this decrease intensifies before reaching a steady state. These data do not support scenario iii, since a post-bleached variation in APG-2 intensity in RB would not be expected if K^+^ flux into the RB and IL were independent. Consequently, these data show that K^+^ is first exchanged between the cytoplasm and the RBs and then between the RBs and the IL, identifying the RB as a master regulator of the inclusion [K^+^] (Figure 7E).

### 3.6. A Role for K^+^ in Bacterial Communication

**Figure 8:**
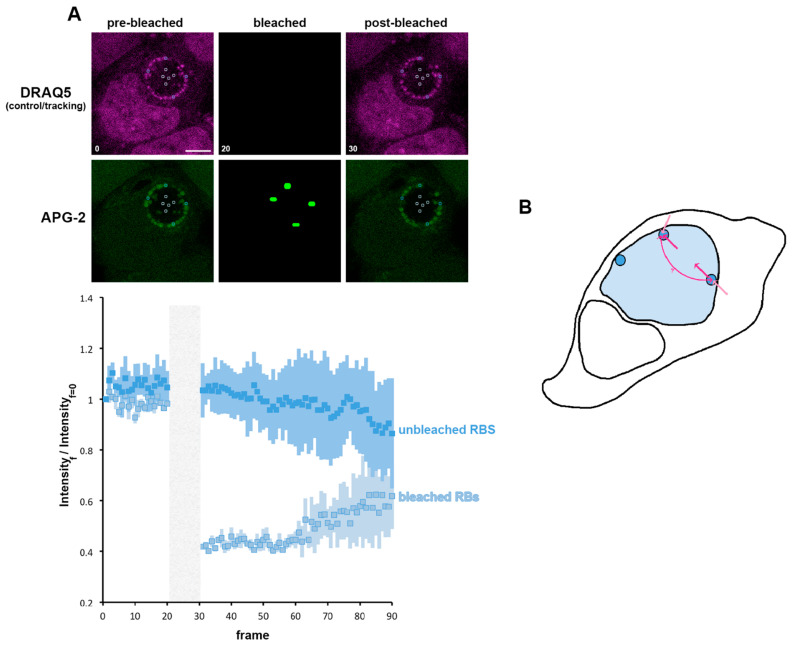
K^+^ is exchanged between reticulate bodies. (**A**) HeLa cells were infected with *C. trachomatis* LGV2. At 24 hpi cells were labelled with APG-2 and DRAQ-5 and then imaged. APG-2 was photobleached as indicated on the upper panels in some reticulate bodies (RBs). Circles show the region of interest used for the analysis of the intensity depending on the frame (lower panels: 1.509 s/frame) presented on the lower panels. Dark blue: unbleached RBs and light blue: bleached RBs. Scale bar: 5 µm. (**B**) Shows schematics of the proposed K^+^ flux (pink arrows), between the host cytoplasm (white) the inclusion lumen (light blue) and the bacteria (dark blue).

We have shown the importance of K^+^ for the RB, and we propose that the RB is the principal regulator of the K^+^ distribution in the inclusion. We now investigated whether transfer of K^+^ between RBs within the inclusion occurred. For this, we bleached individual RBs and observed the subsequent behaviour of both the bleached and unbleached RBs (localised either proximally or distally to the bleached RB) (Figure 8A). The bleached bacteria first show a minimal recovery similar to that observed in Figure 7B. However, the steady state reached after this first recovery does not stabilise, and an important phase of recovery can subsequently be observed. During the bleaching of individual RBs, the non-bleached bacteria do not exhibit any immediate post-bleach modification of APG-2 intensity. However, we observed a decrease in this intensity that mirrors the recovery of the bleached RBs. This demonstrates an exchange of K^+^ between RBs, supporting the hypothesis of inter-bacteria communication within the inclusion supported by K^+^ flux.

### 3.7. A Role for K^+^ in Bacterial Differentiation

**Figure 9:**
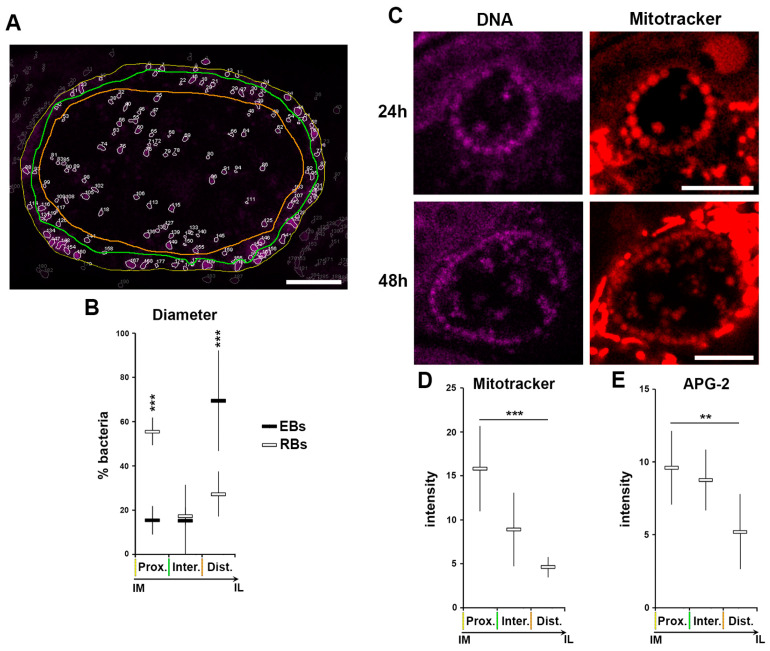
Loss of K^+^ is associated to loss in membrane potential during RB to EB differentiation. HeLa cells were infected with *C. trachomatis* LGV2. All measurements have been performed on inclusions at 48 hpi. (**A**) An example of the segmentation experiments. The edge of the inclusion is highlighted with a yellow line, the region where the bacteria are in close proximity to the inclusion edge is outlined in green, and the luminal space of the inclusion by an orange line. Scale bar: 5 µm. (**B**) Bacterial diameter has been determined and a threshold applied to differentiate the elementary bodies (EBs), reticulate bodies (RBs) and intermediate bodies (IBs) (Figure 2E–H). The relative number (as a percentage) of each bacterial form is plotted against the position in the inclusion following the parameters established in previous panel (Prox.: directly proximal to the inclusion membrane, Inter: intermediate space, Dist.: distal to the inclusion membrane (luminal space). Coloured lines mirror those shown in previous panel. ***: *p* < 0.001. (**C**) At the indicated time point cells were incubated with Mitotracker and DNA probe (DRAQ-5) prior to imaging. Scale bar: 5 µm. (**D**) Mitotracker intensity was determined for RBs in different regions of the inclusion defined in the previous panels (**A** and **B**) ***: *p* < 0.001. (**E**) APG-2 intensity was determined for RBs position in different regions of the inclusion as in previous panel (**A** and **B**) **: *p* < 0.1, ***: *p* < 0.01.

We show that RBs lose K^+^ during their differentiation into EBs, which equilibrates with the inclusion lumen. In itself this constitutes a drastic change in bacterial [K^+^], and provides the opportunity to elucidate the role of K^+^ in the bacteria without the need for induced modification of the cytoplasmic [K^+^] by exogenous stimuli like ionophores or persistence inducers. K^+^ is involved in a plethora of processes, of which a critical one is the modulation of membrane potential. Indeed, in prokaryotic organisms K^+^ fluctuation is able to modify the electric membrane potential [44]. Consequently, we investigated whether during the process of differentiation from RB to EB, when the [K^+^] decreases in the bacterial cytosol, also induces a decrease in the bacterial membrane potential. In common with all probes derived from the TMRE (tetramethylrhodamine, ethyl ester), mitotracker is a cell permeant positively-charged probe that accumulates in membrane charged compartment, such as active mitochondria, and is therefore a commonly used fluorescent tracker for membrane potential. It has been proposed that during differentiation, RBs detach from the inclusion membrane and are then found deeper in the luminal space [13,35]. To experimentally verify this postulate, we applied the same segmentation depicted in Figure 2E–H and Figure S1B using the assigned number-to-particle to define the location of bacteria within three regions—immediately proximal to the inclusion membrane (prox.), inclusion lumen (dist.) and a short intermediate zone (inter.) (Figure 9A). After extraction of the diameter of each particle, we applied a size filter (Figure 2F,H) to identify EBs and RBs, and to simultaneously position them spatially respective to the three defined regions (Figure 9A,B). These data confirmed the displacement of RBs from the inclusion membrane into the lumen as they differentiated into EBs, demonstrating a correlation between bacterial differentiation state and their position within the inclusion.

HeLa cells were infected with *C. trachomatis* LGV2 and labelled with Mitotracker or APG-2 at 24 or 48 hpi prior to confocal live cell imaging (Figure 9C). Bacteria were clearly labelled at both time points, in addition to the cellular mitochondria, demonstrating that membrane potential can be detected in intracellular *Chlamydia* using Mitotracker in live cells. Strikingly, at both time points the RBs attached to the inclusion membrane exhibited the highest signal intensity. Moreover, some bacteria with a diameter in the RB range present in the inclusion lumen also retained the Mitotracker signal. The intensity of Mitotracker was measured in RBs and correlated with the respective position of the bacteria in the inclusion (Figure 9D). This clearly demonstrated that RBs associated with the inclusion membrane (imRBs) have a higher Mitotracker intensity compare to those in the inclusion lumen (ilRBs), showing that imRBs have a higher membrane potential than ilRBs. Interestingly, when the same analysis was applied to APG-2 labelled RBs, a similar profile was obtained (Figure 9E), strongly suggesting a correlation between [K^+^], membrane potential and bacterial differentiation state.

## 4. Discussion

After careful validation, we were able to employ live confocal imaging to visualise the location of K^+^ in *Chlamydia* infected cells. A related approach was reported by Grieshaber and colleagues [45], which concluded that the [K^+^]_inclusion_ is similar to [K+]_cytoplasm_. However, the probe and microscopy applied only allowed the measurement of [K^+^]_whole inclusion_ (EB, RB and IL) as well as its immediate environment by epifluroescence microscopy. In our case, we applied confocal imaging allowing better resolution of the regions of interest. We clearly demonstrated that RBs accumulate K^+^ leading to an average 2.5 times higher intensity of APG-2 than the cytoplasm, which is consistent with the expected [K+] ratio between eukaryotes and prokaryotes [17,46]. We also demonstrated that the APG-2 intensity of the inclusion lumen is ~50% lower than the cytoplasm and ~5 times less than RBs, which is entirely consistent with the data of Grieshaber and colleagues concluding the *average* [K^+^]_inclusion_ is similar to the [K+]_cytoplasm_.

Intriguingly, we demonstrated that RBs scavenge K^+^. Indeed, we observed that while the inclusion contains a mixed population of RBs, EBs and intermediate bodies, newly formed EBs do not accumulate the APG-2 probe, while remaining permeable to a DNA probe. This illustrates that one step in the differentiation of RB into EB involves a loss of K^+^. Considering that RBs must reduce their volume by 75% during transition into EBs, and that the decrease in [K^+^] is associated with reduction in cell volume [47,48], we can hypothesise that the reduction in cytoplasmic K^+^ is a critical step in this transition. Nevertheless, the efflux of K^+^ from the RBs consequently creates an influx in another compartment. It is yet to be determined which compartment is the recipient and how there is a compensation for this change in osmolarity. It should also be considered that this K^+^ influx triggers further signalling, such as the initiation of egress. It is intriguing that cytoplasmic K^+^ efflux is required during productive chlamydial infection to activate caspase-1 [20]. How this might coincide with the loss of K^+^ during RB-EB transition should now be investigated further.

As RBs actively accumulate a high concentration of K^+^ and are abundant in an inclusion, it is interesting to observe that the [K^+^]_cytoplasm_ of the infected cells is not affected when compared to non-infected cells. It is, therefore, unclear whether *Chlamydia* actively influences the regulation of host cytoplasmic [K^+^]. However, understanding the maintenance of host K^+^ osmolality is critical, as K^+^ is involved in the maintenance of membrane potential, cell excitability, turgor pressure, maturation of certain cytokines, the induction of apoptosis, as well as proliferation, cell migration and angiogenesis [49].

As mentioned, K^+^ is important to maintain the membrane potential of eukaryotic cells. In prokaryotes, the membrane potential is primarily a consequence of the proton motive force (PMF) generated by the action of the electron transport chain [50]. This PMF is used in energy production and the transport of nutrients [51]. However, proton movement is associated with other ion flux such as Na^+^ and K^+^ and it is now clear that K^+^ can modulate the proton-generated membrane potential [14,44]. *Chlamydiae* only possess an incomplete electron transfer chain [52], which could have consequences for the PMF and therefore the proton gradient and membrane potential. The membrane potential of *Chlamydia* has never been measured or observed. We observed this by exploiting the properties of Mitotracker and demonstrate that indeed, *Chlamydia* exhibits a membrane potential. Interestingly, we were able to show that the membrane potential varies with the position of the RB, the bacteria being more energised in close apposition with the inclusion membrane. Intriguingly, we observed the same decrease in the APG-2 intensity depending on the position of the RB within the inclusion. While more mechanistic investigation is now required, we can conclude that there is a strong correlation between the bacterial differentiation state, the membrane potential and the bacterial [K^+^].

To demonstrate specificity for K^+^, we used different ionophores to define that the *Chlamydia* life cycle is not sensitive to a variation in the [Ca^2+^] or [Cl^−^]. Since, to date, no ionophores are selective for either K^+^ or Na^+^, an additional role for Na^+^ cannot be unequivocally excluded using ionophores alone. Consequently, to circumvent such lack of specificity, and confirm the role of K^+^ in the *Chlamydia* lifecycle we additionally employed glibenclamide, a drug specific for K^+^ channels, in combination with APG-2. This showed that interfering with accessible host cytoplasmic [K^+^] similarly leads to chlamydial persistence. Persistence is a bacterial response to stress, which requires sensing mechanisms. How *Chlamydiae* sense the variation in [K^+^] in the host cell is an open question—three scenarios are plausible: (i) the bacteria translocate a K^+^ sensor into the cytoplasm that is able to initiate a cascade to transduce K^+^ through the inclusion membrane into the RB cytoplasm, (ii) the bacteria possess a voltage gated K^+^ channel at the RB/cytosol interface or (iii) the stimuli directly lead to a change in the RB membrane potential which induces persistence. At present, all these scenarios are equally valid, scenario (i) would imply the involvement of a prokaryotic sensor which has been identified in *E. coli* [53]; scenario (ii) would highlight the RB/cytoplasm interface that we demonstrated to be important; and scenario (iii) is based on the importance of the maintenance of the membrane potential which we demonstrated to be regulated during RB to EB transition, and has been shown to be critical for the control the prokaryotic cell division process [54], which is intriguing as cell division arrest is a trigger for *Chlamydia* persistence [3].

Finally, we observed a very interesting exchange of K^+^ between RBs, allowing us to speculate that K^+^ could be used as a means to pulse communication between bacteria allowing synchronicity and quorum behaviour within the inclusion.

## Supplementary Materials

The following are available online at https://www.mdpi.com/2076-2607/9/1/173/s1, Figure S1: Analysing K+ accumulation and flux in live cells, Figure S2: The requirement for K+ is independent of host cell type and bacterial strain and species, Figure S3: Characterisation of the glibenclamide treatment and kinetics of nigericin activity.
