# Supplementary material for: Chlamydia Uses K+ Electrical Signalling to Orchestrate Host Sensing, Inter-Bacterial Communication and Differentiation
Source: Microorganisms. 2021 Jan 15;9(1):173. doi: 10.3390/microorganisms9010173 (PMC7830353; doi:10.3390/microorganisms9010173)
Supplement: Supplementary file 1 [file microorganisms-09-00173-s001.pdf]

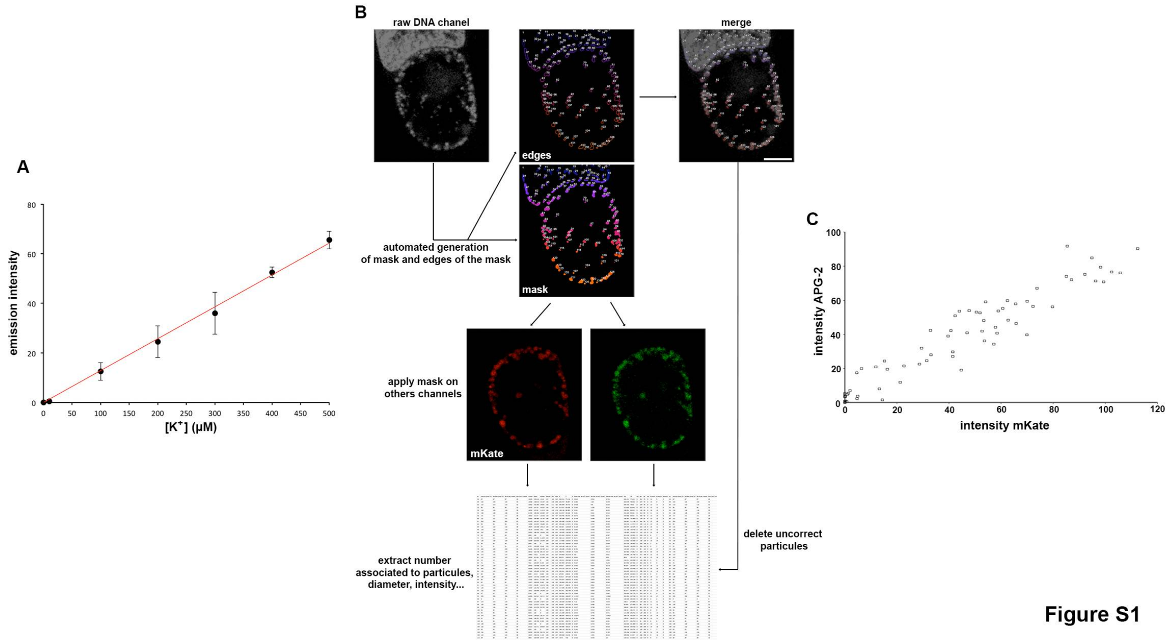

Figure S1

Figure S1: Analysing K^+ accumulation and flux in live cells. (**A**) standard curve used during flame photometry experiments. On the x-axis, [K^+] in deionised water. On the y-axis, the emission intensity. (**B**) Segmentation pipeline. Using ImageJ, as previously described (Dumoux 2012) the DNA channel is subjected to segmentation with the 'watershed' algorithm being applied to the binary image prior to particle isolation. The generated mask is then applied to the channel to be analysed and the intensity of the generated ROIs measured as previously described (Dumoux 2012). The generated list is manually curated and the position of the particle can located by the reference number associated with each element. Scale bar: 5 µm. (**C**) HeLa cells were infected with *C.trachomatis* LGV2 transformed with pASK-GFP-L2 allowing the expression of mKate (red) only when the bacteria are in the RB form. Using the approach mentioned in panel B, mKate and APG-2 intensities were measured for each individual bacteria, and plotted against each other to determine the correlation factor.

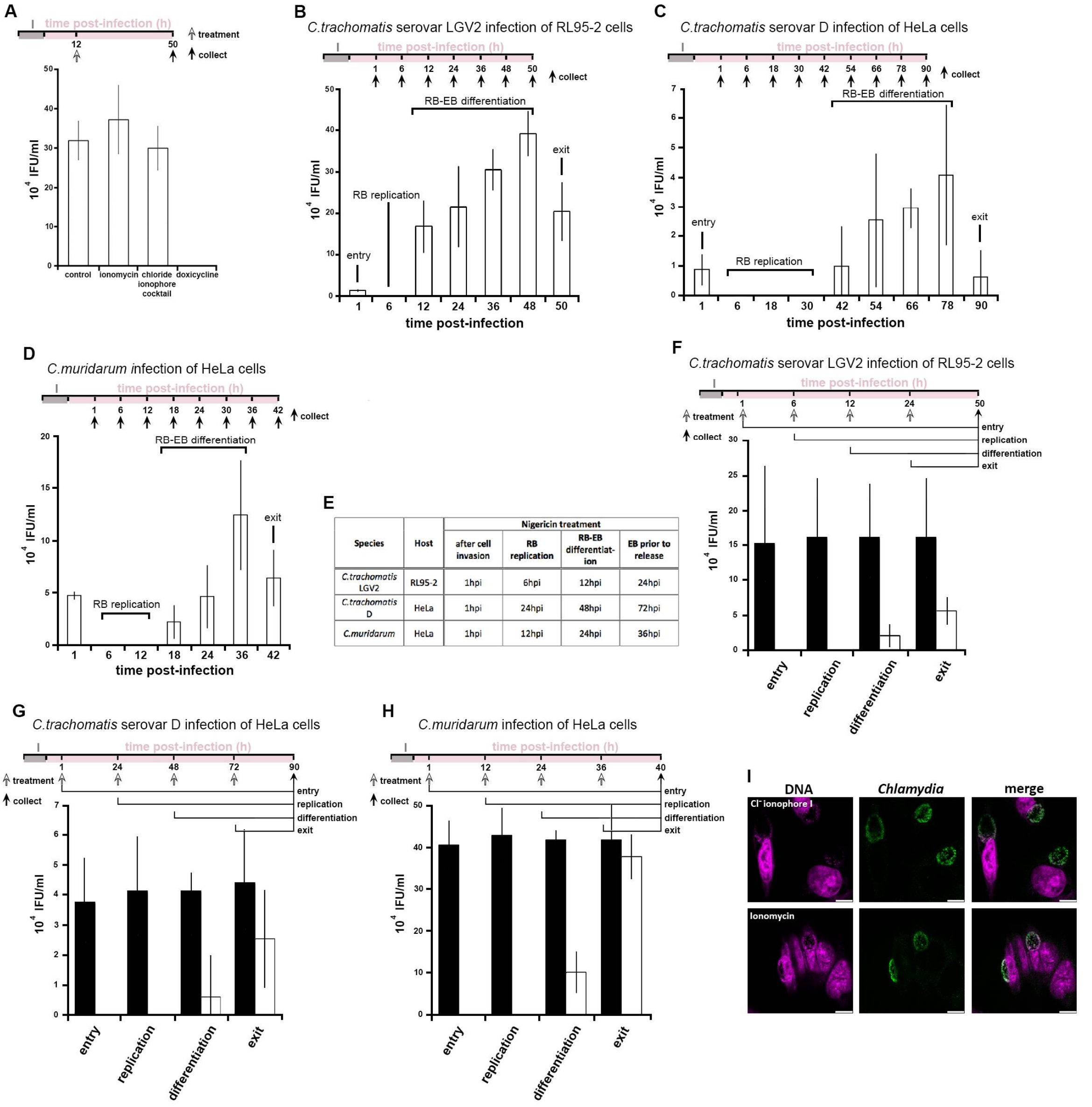

E

Species	Host	Nigericin treatment			
		after cell invasion	RB replication	RB-EB differentiat-ion	EB prior to release
C.trachomatis LGV2	RL95-2	1hpi	6hpi	12hpi	24hpi
C.trachomatis D	HeLa	1hpi	24hpi	48hpi	72hpi
C.muridarum	HeLa	1hpi	12hpi	24hpi	36hpi

Fig S2: The requirement for K^+ is independent of host cell type and bacterial strain and species. (**A**) top panel shows the experimental design. HeLa cells were infected with C.trachomatis LGV2 and treated as indicated. After sample collection, infectivity assays were performed (IFU/mL: inclusion forming unit per mL). (**B to D**) cells were infected (I) with the indicated bacteria strain/species. Cells were collected as indicated by the black arrows and the infectivity assayed allowing the identification of the different phases of the lifecycle –entry, reticulate body (RB) replication, RB-elementary body (EB) differentiation, exit. (**E**) The infectivity assay allows infection cycle progression to be characterised using different combinations of bacterial species and host cell type. (**F to H**) Upper panels show the experimental design: cell types, as designated, were infected as with the specified species of *Chlamydia*, and treated with nigericin at the appropriate time to interfere with the different phase of the bacteria lifecycle (panel B to E). Cell samples were then collected at the appropriate timepoint and infectivity assays performed. Lower panel show the infectivity assay performed for the different

conditions. (I) HeLa cells were infected with *C.trachomatis* LGV2 and treated at 12 hpi with chloride (Cl^-) ionophore or ionomycin (calcium ionophore). At 24 hpi, cells were fixed prior to labelling with DNA probe (DRAQ-5, magenta) and *Chlamydia* (green). Scale bar: 10 μm.

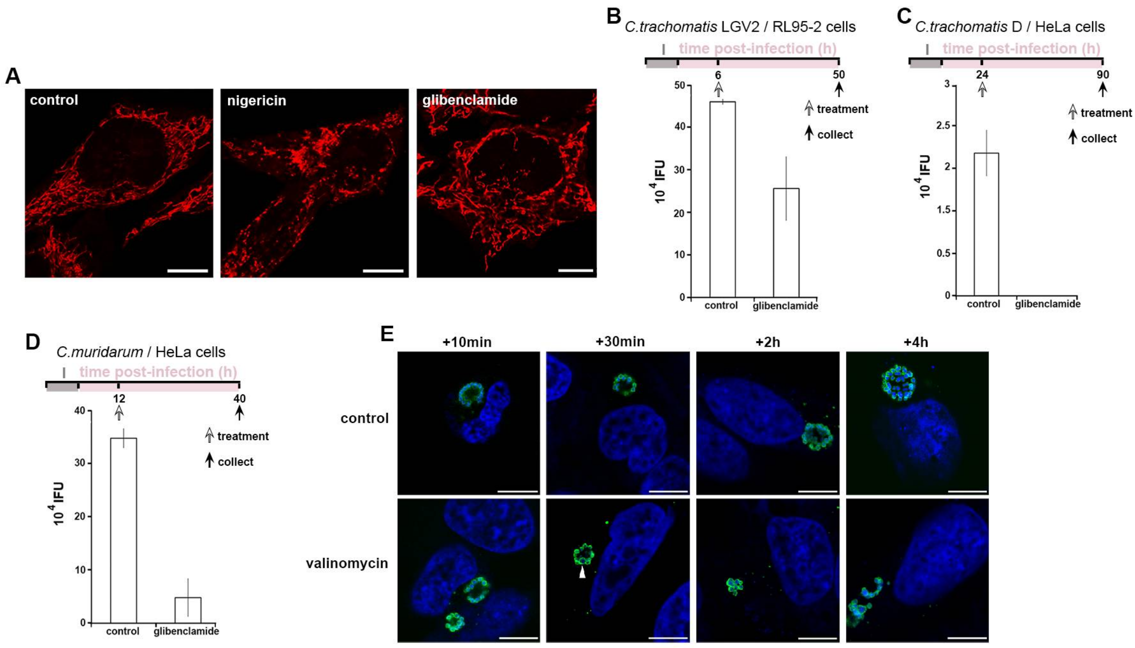

Figure S3: Characterisation of the glibenclamide treatment and kinetics nigericin activity. (**A**) HeLa cells were treated with nigericin or glibenclamide 12 h prior to incubation with Mitotracker (red) followed by fixation and imaging. Scale bar: 10 μm. (**B to E**) HeLa cells were infected with *C.trachomatis* LGV2. (**B to D**) upper panel show the experiment design. Cell types, as designated, were infected with the specified species of *Chlamydia*, and treated with glibenclamide at the appropriate time to interfere with the RB replication phase (**Figure S2 B to E**). At the appropriate time, cell samples were collected and infectivity assays performed. Lower panels show the infectivity of the different species of *Chlamydia* grown in the different cell lines in the presence or absence of glibenclamide (IFU/mL: inclusion forming unit per mL). (**E**) At 12 hpi, HeLa cells infected with *C.trachomatis* LGV2 were treated with valinomycin or not and fixed at the indicated time and labelled for *Chlamydia* (green) and DNA (DRAQ-5, blue), scale bar: 10 μm.
